# A comparative analysis of multi-stage evaporative cooling and conventional chillers for office-scale cooling loads

**DOI:** 10.1038/s41598-026-41650-9

**Published:** 2026-04-02

**Authors:** C. Chiranjeevi, Y. Raja Sekhar, J. Javith, K. Dilip Kumar, Muhammad Asif, Mohamed Bechir Ben Hamida, Gabr Goshu Syum

**Affiliations:** 1 School of Mechanical Engineering, Vellore Institute of Technology, Vellore, TN 632014 India; 2 Environment and Sustainability Global Sustainability Solutions and Services QFZ LLC (GSustain), Doha, Qatar; 3 Nitte (Deemed to be university), NMAM Institute of Technology, Nitte. Department of Mechanical Engineering., Mangalore, 575018 India; 4 Department of Mathematics and Statistics, College of Sciences, Imam Mohammad Ibn Saud Islamic University (IMSIU), Riyadh, Saudi Arabia; 5 Deanship of Scientific Research, Imam Mohammad Ibn Saud Islamic University (IMSIU), Riyadh, Saudi Arabia; 6 Centre of Studies in Oil and Gas Engineering and Technology (CS-OGET), Eduardo Mondlane University, Moçambique, Bairro Luís Cabral, Maputo, Mozambique; 7 Faculty of Mechanical and Industrial Engineering, EiT-M, Mekelle University, P. O. Box 231, Zip Code: 7000 Mekelle, Tigray Ethiopia

**Keywords:** Evaporative cooling, Air psychometric, Cooling effectiveness, Energy efficiency

## Abstract

The building sector accounts for a substantial share of global electricity consumption, with HVAC systems dominating energy use in large commercial buildings, particularly in developing countries. This study presents a comparative, climate-specific performance assessment of a hybrid multi-stage evaporative cooling–chiller system, with a specific focus on modifying an indirect evaporative cooling (IEC) unit by utilizing return air as the working air stream which is a configuration rarely evaluated for Indian climatic conditions. The methodology combines psychrometric analysis, real-time system modification, and detailed building heat-load calculations for a large office-scale building across multiple Indian climate zones under summer and monsoon conditions**,** and benchmarks three configurations: (i) stand-alone chiller, (ii) conventional IEC–chiller system, and (iii) modified IEC–chiller system. Results show that the conventional IEC configuration reduces chiller capacity by 50–60 TR**,** while the modified multi-stage IEC system achieves a chiller capacity reduction of 80–140 TR, depending on climate and seasonal load. For identical cooling demands, the conventional IEC system consumes 27.53% less electrical energy than a stand-alone chiller, whereas the modified IEC–return air configuration delivers a higher reduction of 34.97%. The modified system also demonstrates superior temperature reduction and wet-bulb effectiveness, particularly under humid monsoon conditions where conventional IEC performance is typically constrained. These findings demonstrate that return-air-assisted multi-stage evaporative cooling significantly enhances chiller load reduction and energy efficiency, offering a technically viable and scalable hybrid cooling solution for large buildings in diverse Indian climates.

**Supplementary Information:**

The online version contains supplementary material available at 10.1038/s41598-026-41650-9.

## Introduction

In tropical non-coastal regions, climatic conditions are typically hot and dry, resulting in a high demand for space cooling and substantial energy consumption. In many developing countries, this demand is still largely met by coal-based electricity, contributing significantly to environmental degradation and carbon emissions^1^. Climate change has further accelerated the deployment of HVAC systems in commercial buildings, where the widespread use of high-GWP refrigerants and energy-intensive vapour compression technologies continues to increase reliance on fossil fuels. As a result, conventional HVAC systems exhibit poor sustainability when evaluated in terms of energy use per unit of cooling delivered. Achieving global targets such as net-zero emissions by 2050 therefore necessitates the development and adoption of energy-efficient and environmentally benign cooling technologies.

Multi-stage evaporative cooling systems present a promising alternative, combining indirect and direct evaporative cooling with a conventional chiller coil to reduce overall energy demand while maintaining thermal comfort across diverse climatic conditions. The integration of a chiller coil is particularly advantageous during the Indian monsoon season, where high humidity and low wet-bulb depression significantly limit the effectiveness of evaporative cooling alone. Evaporative pre-cooling reduces chiller load, enabling higher outdoor air intake without additional energy penalties and improving indoor air quality through increased air changes per hour. In indirect evaporative cooling, sensible cooling occurs at nearly constant humidity, as evaporation is confined to the secondary air stream, which is exhausted to the ambient.

Multi-stage evaporative cooling systems demonstrate superior performance compared to conventional HVAC systems, achieving cooling effectiveness of up to 95%, energy consumption as low as 0.3–1.2 kW/t, and COP values reaching 35^2^. These systems can reduce dry-bulb temperature by nearly 50% and specific humidity by up to 80%, while offering short payback periods of approximately 3–4 years^3,4^. Their reliance on water instead of synthetic refrigerants significantly reduces environmental impact, making them suitable for residential, commercial, and industrial applications, particularly in hot climates. However, their performance deteriorates under high-humidity conditions, highlighting the need for design modifications and hybrid configurations.

Previous studies have extensively examined indirect, direct, and combined evaporative cooling systems, reporting substantial improvements in cooling effectiveness, energy savings, and thermal comfort across dry and semi-arid regions^5^. Research has also explored advanced configurations incorporating dew-point cooling, desiccant dehumidification, alternative heat exchanger materials, and solar-assisted systems^6^. While these studies confirm the energy-saving potential of multi-stage evaporative cooling, challenges remain related to water consumption, airflow optimization, and performance reliability under humid and mixed climatic conditions, particularly in India.^7^.

**Fig. 1:**
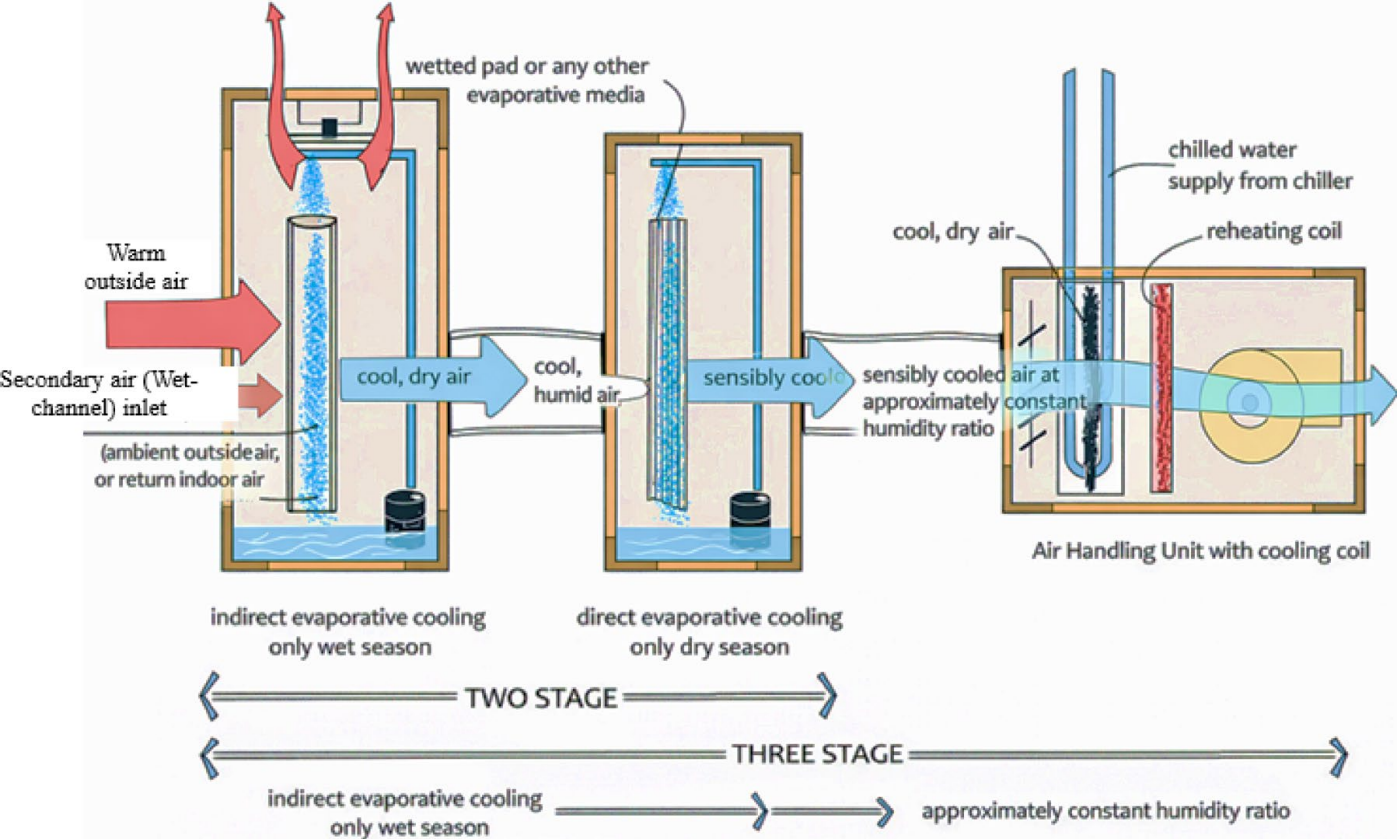
Schematic layout of multi-stage evaporative cooling system^9^.

In tropical non-coastal regions of India, increasing cooling demand continues to drive energy use from non-renewable sources, while conventional vapor compression systems remain environmentally unsustainable. Although multi-stage evaporative cooling systems, as shown in Fig. 1, offer significant advantages, their effectiveness is constrained by low wet-bulb depression during humid periods and limitations in existing IEC designs^8,9^. Notably, the energy efficiency of return-air-based IEC modifications across different Indian climatic zones has not been comprehensively evaluated^4,5^. Recent climate assessments report by IPCC, a significant increase in cooling degree days (CDD) and more frequent extreme heat events, with peak dry-bulb temperatures in many urban regions rising by 1–2 °C over the past three decades. These shifts have directly increased space cooling demand in commercial buildings^10^.

There is an apparent information gap in the literature on the experimental validation of return air-assisted indirect evaporative cooling systems, especially when such systems are used in combination with conventional chillers in large commercial buildings in India. Although from past studies it is understood that direct, indirect, and multiple-stage return air-assisted IEC systems are effective in conserving energy in dry and semi-arid climates, the majority of the past research has relied on theoretical evaluations or pilot plant studies^11–17^. There is a need to conduct a detailed performance evaluation of the return air-assisted IEC system in combination with conventional chillers under Indian summer and monsoon weather conditions, and there are few studies that quantify the improvement or degradation of the capacity of chillers, the energy savings, and the wet bulb temperature effect of such systems under multiple climatic zones with actual heat load data. Hence, there is a research gap, and experimental validation and benchmarking of a modified return air IEC system with respect to conventional IEC systems and standalone chiller systems, considering various Indian climate zones, is necessary. The objective of this study is to bridge this gap by introducing a real-time modification to the system and performing a comparative analysis with respect to various Indian climate zones. The study also includes a psychometric analysis and precise calculations for determining the building heat load to estimate how much reduction is possible in chiller capacity and how much energy can be saved.

## Description of indirect evaporative cooler (IEC) system

### Working principle of indirect evaporative cooler

**Fig. 2:**
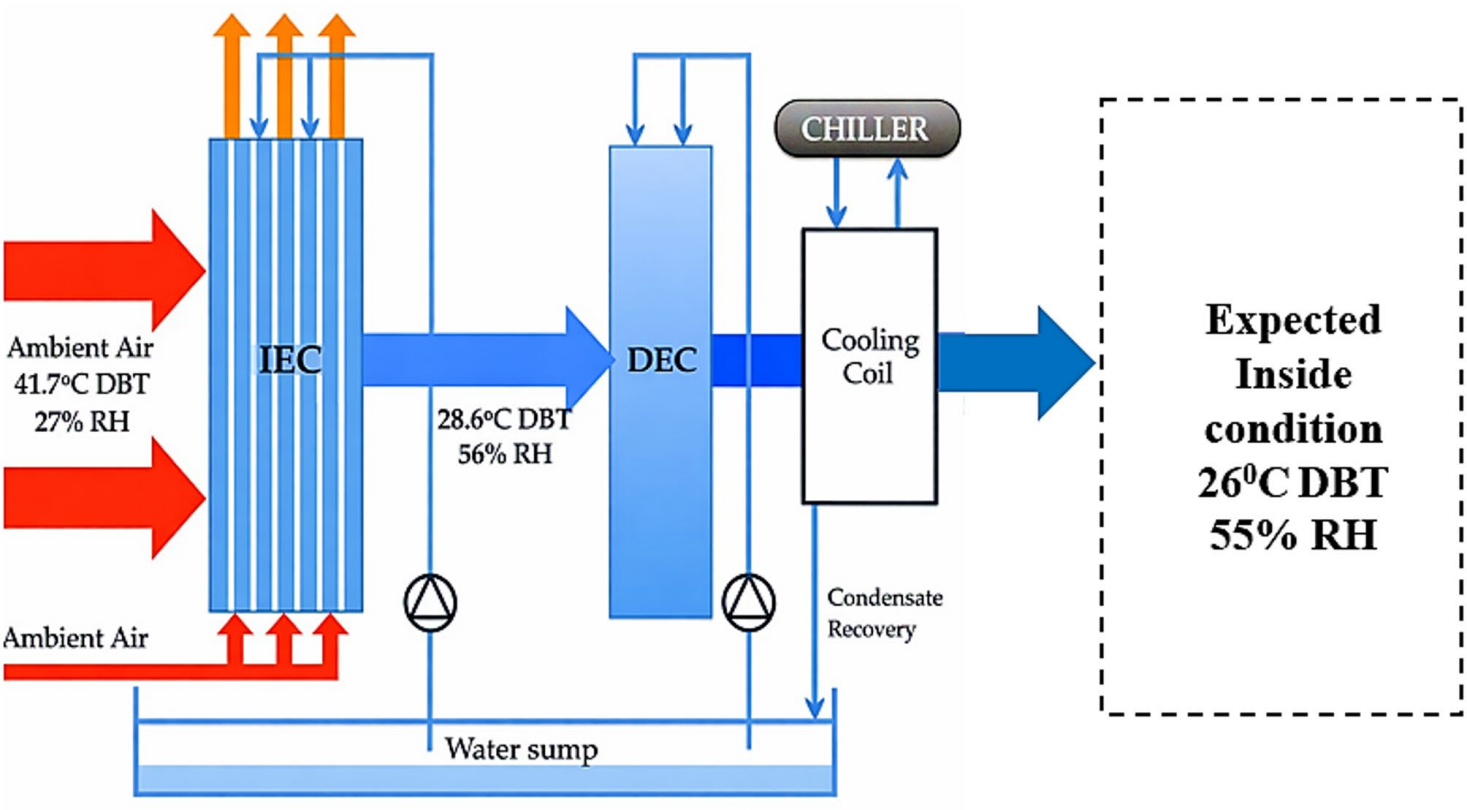
Schematic layout of the conventional indirect evaporative cooling system^18^.

Indirect Evaporating Cooler (IEC) system, as presented in Fig. 2, cools air without adding moisture, making it more suitable than Direct Evaporative Cooling (DEC). In IEC, primary air passes through a dry channel, while secondary air moves through a wet channel. The heat from the primary air is absorbed via water evaporation in the wet channel, effectively lowering its temperature through latent heat transfer. For instance, when an air stream at a dry bulb temperature of 41.7 °C and 27% relative humidity passes through the existing indirect evaporative cooling unit, the outlet dry bulb temperature reduces to 28.6 °C, and the relative humidity increases to 56%. The system achieves a wet bulb effectiveness of 80%, indicating that the outlet temperature is 80% closer to the wet bulb temperature the minimum achievable temperature through evaporative cooling.

**Fig. 3:**
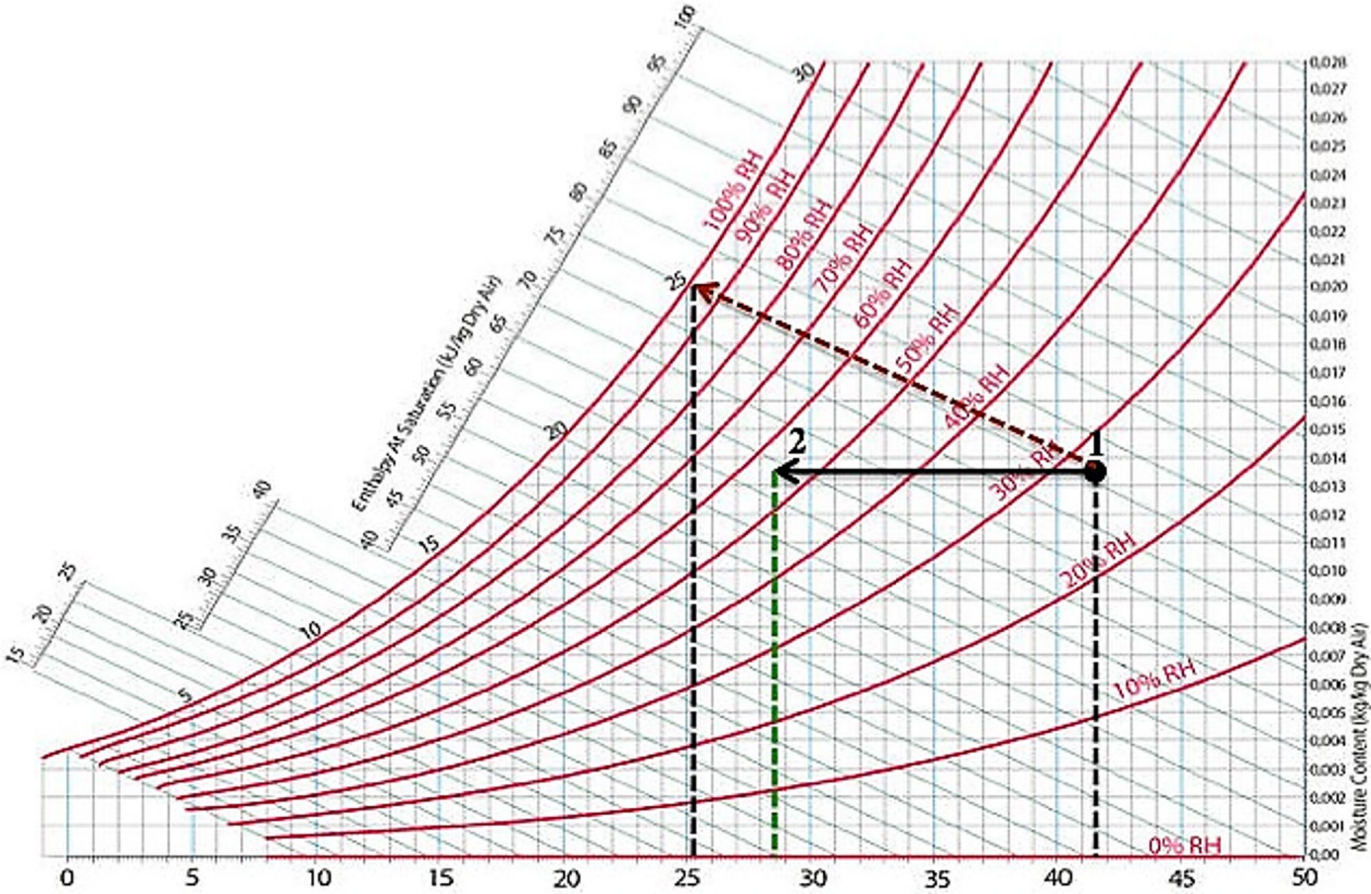
Illustration of the psychrometric chart for conventional IEC operating conditions^18^.

Figure 3 illustrates the psychrometric process of air treatment in the conventional indirect evaporative cooling system. In this system, the product air’s temperature can be lowered from its dry bulb temperature to its wet bulb temperature. However, due to limitations in the heat exchange surface area and uneven water distribution, the actual cooling effectiveness is only 40–80% of the wet bulb temperature of the incoming air. During operation, the primary air is sensibly cooled through the latent heat transfer from the evaporation of water on the wet side of the system, which is then transferred to the secondary air. As a result, the primary air temperature decreases without any increase in its moisture content. Meanwhile, the secondary air becomes gradually saturated, heats up along its path, and is eventually discharged to the atmosphere in a saturated state.

### Wet bulb depression

The Wet Bulb Depression (WBD) refers to the difference between the Dry Bulb Temperature (DBT) and the Wet Bulb Temperature (WBT) of the air stream. WBD is inversely proportional to the relative humidity of the air stream. When the relative humidity reaches 100%, the air is fully saturated, resulting in a zero wet-bulb depression. This means there is no temperature difference between the dry bulb and wet bulb, as the air has reached its maximum moisture-holding capacity.

1 $$WBD = T_{db} - T_{wb}$$

where T_db_ is the dry bulb temperature, and T_wb_ is the wet bulb temperature of air.

The effectiveness of an indirect evaporative cooling system is directly proportional to the wet bulb depression of the incoming air stream. When the wet bulb depression is high, the system can achieve a greater temperature reduction. However, as the relative humidity of the ambient air increases, the wet bulb depression decreases, which in turn reduces the performance of the indirect evaporative cooler. This makes the system less effective in humid climates, where the cooling potential is limited due to the smaller temperature difference between the dry bulb and wet bulb temperatures.

### Improvements to the IEC system for enhanced performance

**Fig. 4:**
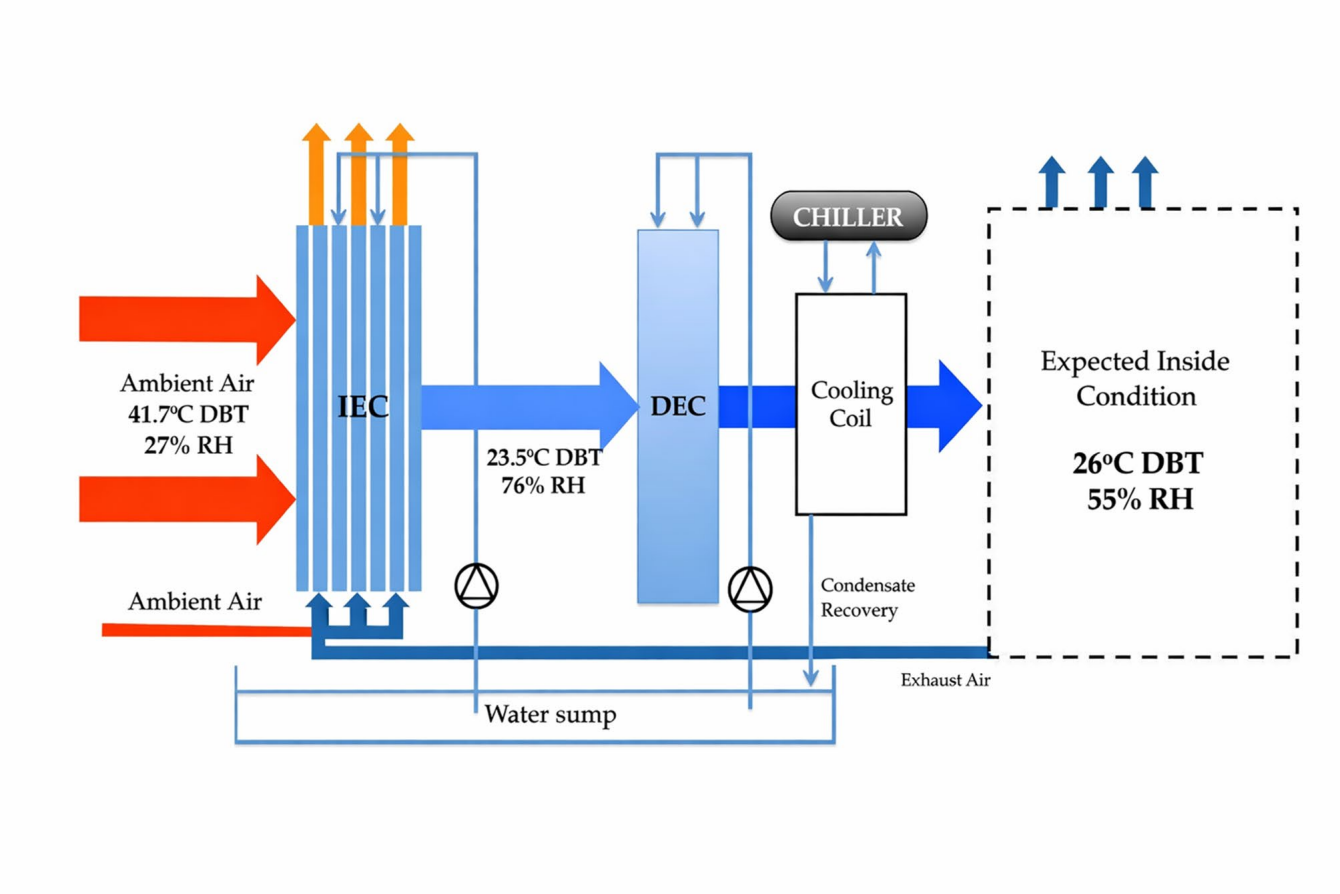
Schematic layout of the modified indirect evaporative cooling system^18^.

To improve the cooling efficiency and overcome the limited temperature reduction of the existing indirect evaporative cooling (IEC) system, the airflow pattern has been modified. Figure 4 shows the modified IEC suitable for humid or moist climates. In the earlier system, ambient air serves as the secondary air, and its properties fluctuate with unstable environmental conditions, leading to inconsistent performance. In humid regions, the high relative humidity significantly reduces the system’s effectiveness.

By using return exhaust air as the secondary air for the indirect evaporative cooling system, these issues are addressed. Return exhaust air from the conditioned space is typically warmer than ambient air due to the building’s heat loads. However, during air conditioning operations to maintain desired temperature and humidity levels, the exhaust air will be cooler than the ambient air. This modification allows for a more stable and effective cooling performance. Additionally, since there is no direct contact between the primary and secondary air streams, indoor air quality concerns, even if poor, will not affect the product air.

Considering an air stream with a dry bulb temperature of 41.7 °C and 27% relative humidity flowing through the existing indirect evaporative cooling unit, the outlet conditions are expected to be a dry bulb temperature of 23.5 °C and 76% relative humidity. The wet bulb effectiveness of the system is calculated to be 111%. This indicates that the cooling system performs beyond the typical limitations, achieving a temperature drop greater than the theoretical wet bulb temperature. When cooled return air is used as the secondary air in the indirect evaporative cooler, the system demonstrates the capability to lower the temperature of the air stream below its wet bulb temperature, surpassing the conventional performance limits of standard IEC systems. This modification enhances the cooling potential, especially in conditions where ambient humidity is a limiting factor. In this study, the monsoon season is defined based on HVAC design outdoor conditions characterized by high relative humidity (typically 75–85%), low wet-bulb depression, and moderate dry-bulb temperatures, as specified in the ISHRAE Design Data Book (2017). These conditions result in a latent-load-dominated cooling demand, making dehumidification the primary challenge for HVAC systems. The monsoon season is therefore critical for HVAC analysis, as conventional evaporative cooling systems exhibit reduced effectiveness under high humidity, and cooling coils experience increased latent load and energy consumption. Evaluating system performance under monsoon conditions is essential to assess the practical applicability and energy-saving potential of hybrid evaporative–chiller systems in Indian climates.

## Performance comparison for different cooling system configurations and heat load calculations

To evaluate the performance of the modified indirect evaporative cooling system, a comparison is made with the existing indirect evaporative cooling system and a conventional stand-alone chiller system. Three system configurations have been developed for this analysis considering an air flow rate of 50.29 kg/s. For simplicity, the air stream bypasses the Direct Evaporative Cooling (DEC) unit in all configurations, ensuring a focus on the indirect cooling aspects.

### Configuration-1: IEC with ambient air as working air

**Fig. 5:**
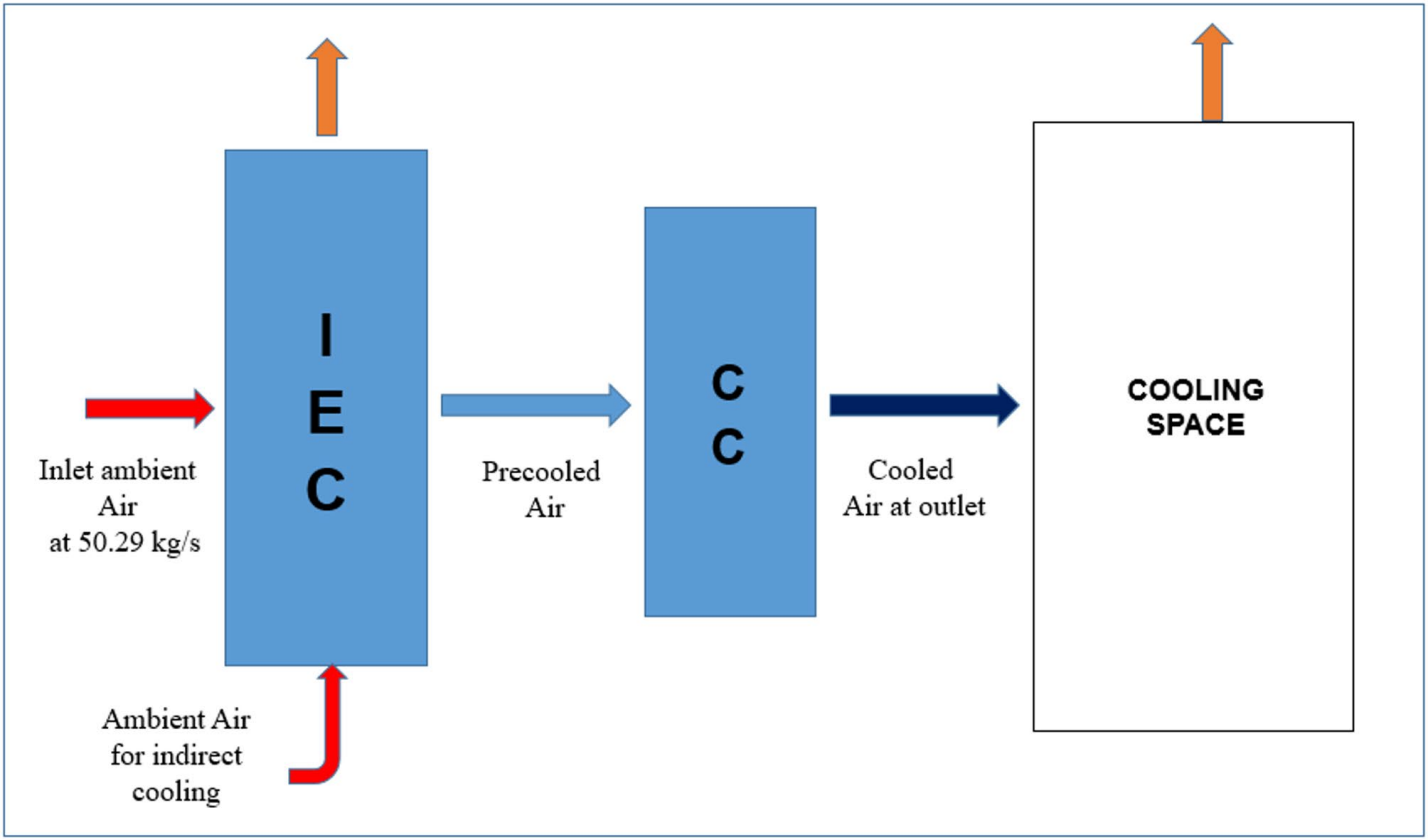
Schematic layout of modified IEC system with ambient air as working fluid.

The first configuration represents the existing indirect evaporative cooling system, where ambient air is used as the secondary (working) air as shown in Fig. 5. In this configuration, the primary air passes through the IEC system, followed by a cooling coil (CC) connected to a chiller that supplies chilled water for further cooling. The exhaust air is expelled to the ambient, with no mixing or regeneration from the exhaust air. This configuration highlights the basic operational principles of the current IEC system without modifications.

### Configuration-2: IEC with return air as working air

**Fig. 6:**
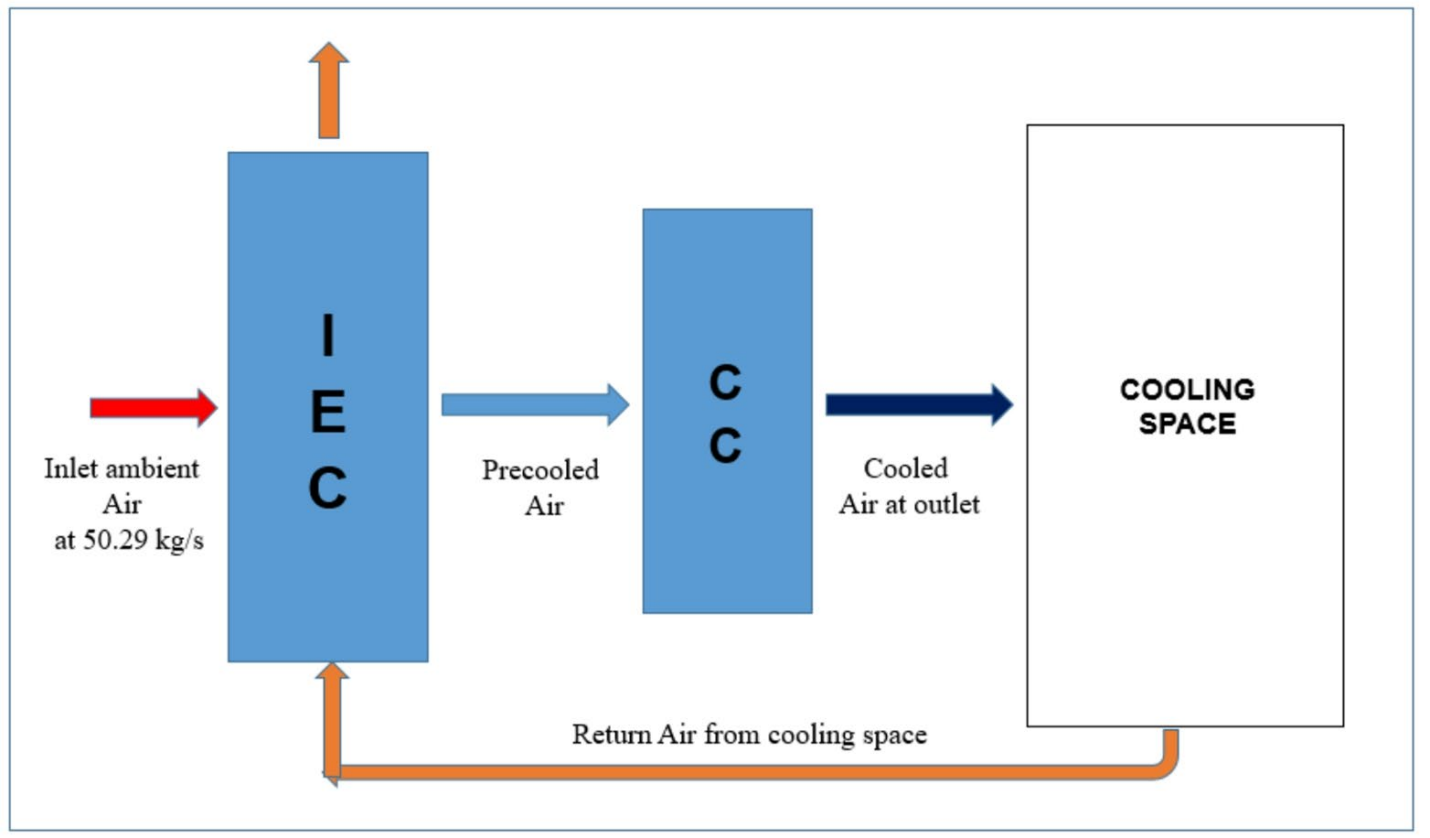
Schematic layout of modified IEC system with return air as working fluid.

The second configuration is the modified indirect evaporative cooling system, where return exhaust air is used as the secondary (working) air as shown in Fig. 6. In this system, the airflow path for primary air remains the same as the first configuration. However, the exhaust air from the cooling space is entirely redirected to serve as the secondary air in the IEC unit and then expelled to the ambient. This modification improves the performance by using warmer, more conditioned return air instead of ambient air, enhancing the cooling potential.

### Configuration-3: stand-alone chiller system

**Fig. 7:**
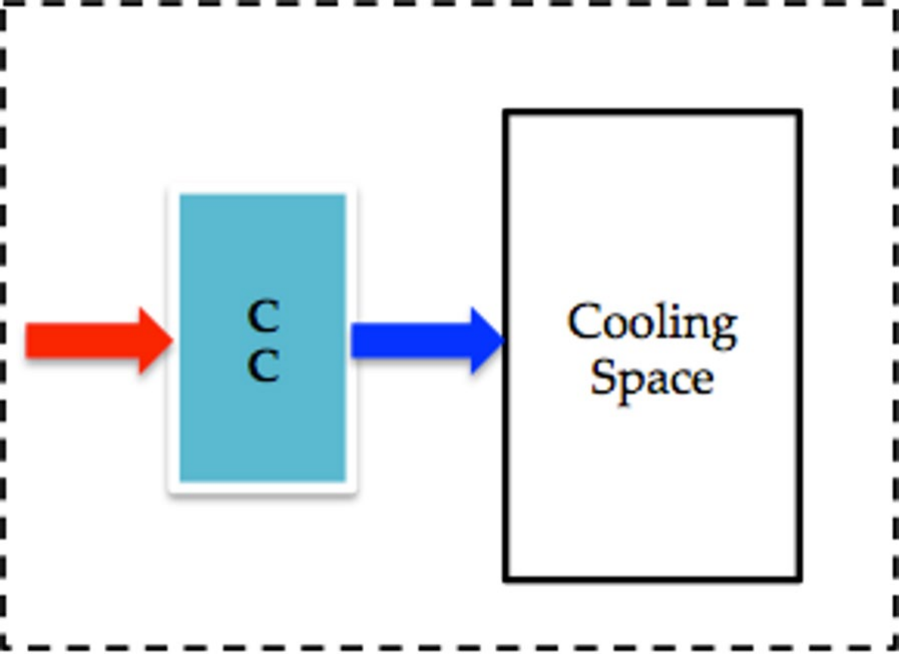
Stand-alone chiller systems.

The third configuration represents the conventional stand-alone chiller system, consisting solely of a chiller coil connected to a chiller unit as shown in Fig. 7. This configuration serves as a benchmark to compare the energy-saving potential of both the existing and modified IEC systems.

### Heat load calculations

**Fig. 8:**
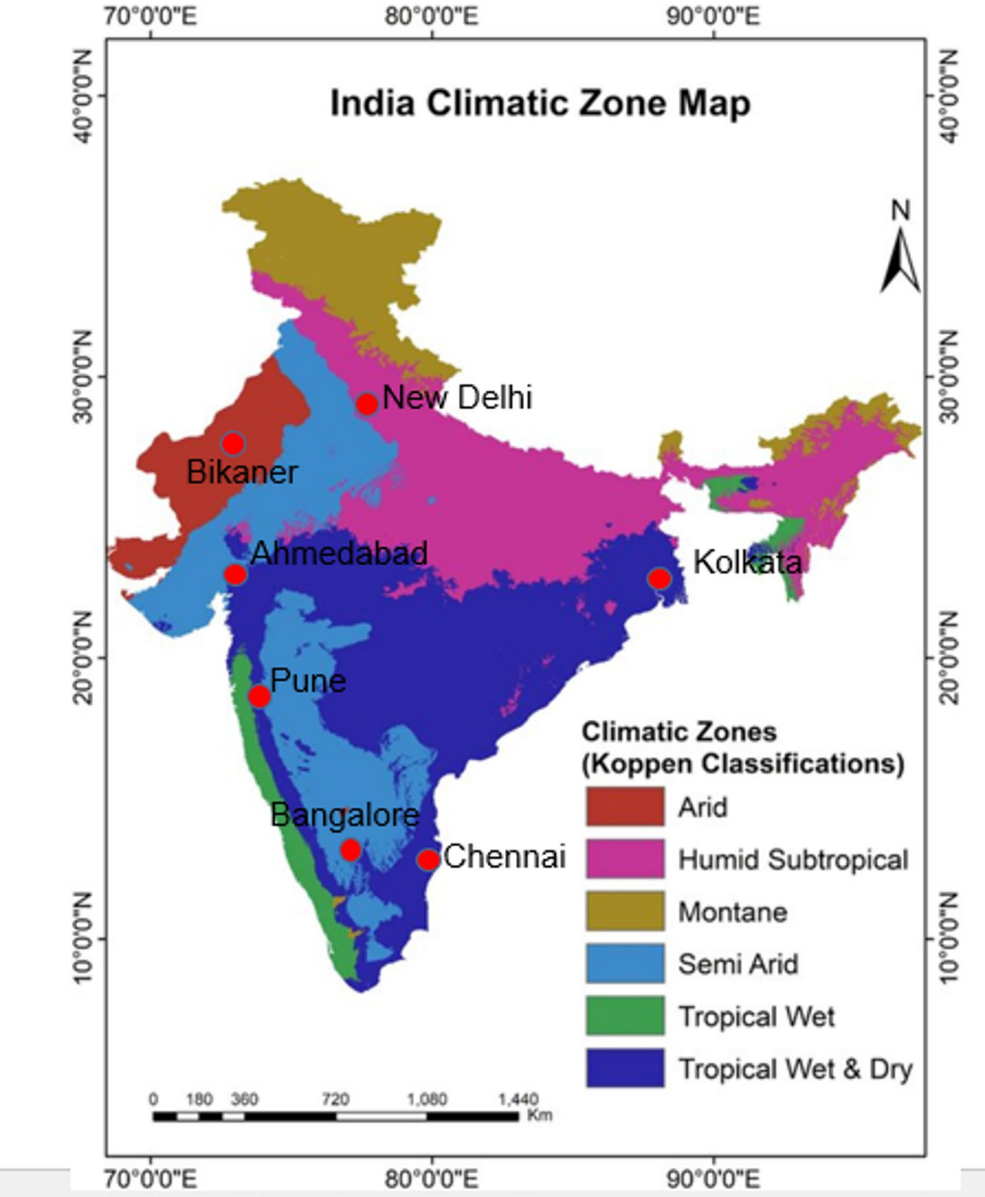
Koppen climatic zones classification and the various cities of India^19^.

As per the Bureau of Indian Standards, the Indian sub-continent is divided into five different climatic zones such as (i) hot and dry (ii) warm and humid (iii) temperate (iv) cold (v) composite. Regions with similar climatic characteristics are grouped and formed as climatic zones as depicted in Fig. 8.

**Fig. 9:**
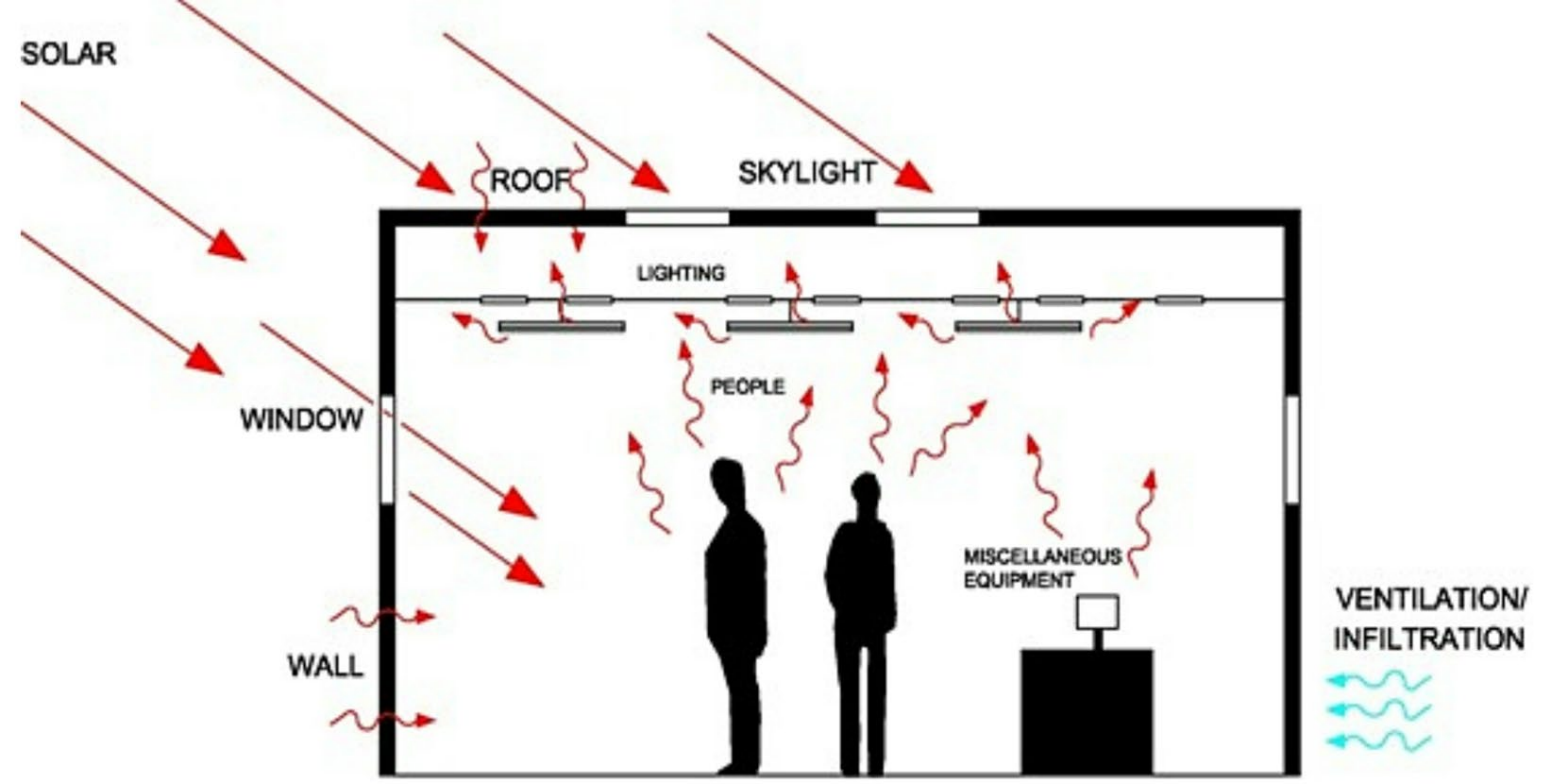
Heat load components in the industrial building^9^.

To assess the comparative performance of the different cooling system configurations in terms of temperature reduction potential for real-world applications, heat load calculations are performed for an industrial building. This industrial building has a shop floor at ground level, divided into two blocks, with an approximate area of 80,950 square feet. The floor height is 18 feet, and it accommodates 150 occupants with a connected load of 380 kW. The heat load of the building consists of sensible heat gains through the roof, walls, and windows; internal heat gains from people, lighting, and equipment; and outdoor heat gains through infiltration and ventilation as shown in Fig. 9.

The heat load calculations for both summer and monsoon seasons are based on outdoor design conditions provided in the Indian Society of Heating, Refrigerating and Air Conditioning Engineers (ISHRAE) Design Data Book (2017). The indoor design temperature is set to 27 ± 1 °C with a relative humidity of 55 ± 5%. The heat load calculation for the selected industrial building under summer design conditions and for the monsoon season are available in Tables A1, A2 respectively and provided as supplementary files. All necessary parameters for the calculations have been sourced from the ISHRAE Design Data Book 2017 and applied as per standard practices.

For the analysis, different cities located in varied climatic zones of India are chosen and relative outdoor conditions for summer as well as monsoon are presented in Tables 1 and 2 respectively. The values of parameters presented in Tables 1 and 2 are provided as input for three cooling system configurations.

**Table 1:** Outdoor design conditions in different cities for summer as per ISHRAE.

Outside design conditions (Summer)					
Location	Mean sea level (m)	DBT (°C)	RH (%)	WBT (°C)	W (g/kg)
Ahmedabad	55	42.10	20.00	23.40	10.5
Bikaner	224	44.40	11.00	21.00	6.4
Chennai	16	38.40	36.00	25.50	15.3
Kolkata	6	37.10	44.00	26.60	17.7
Bangalore	921	34.20	27.00	19.80	10.2
Pune	559	38.10	36.00	25.11	16.2
New Delhi	216	42.00	17.00	22.10	8.9

**Table 2:** Outdoor design conditions in different cities for monsoon as per ISHRAE.

Outside design conditions (Summer)					
Location	Mean sea level (m)	DBT (°C)	RH (%)	WBT (°C)	W (g/kg)
Ahmedabad	55	30.90	82.00	28.27	23.5
Bikaner	224	30.30	81.40	27.58	23.0
Chennai	16	32.10	82.90	28.40	23.1
Kolkata	6	32.40	80.00	29.14	24.6
Bangalore	921	25.30	84.00	23.15	19.1
Pune	559	26.17	84.00	24.02	19.3
New Delhi	216	30.70	82.00	27.99	23.6

Based on the outdoor conditions prevailing at different regions considered for the analysis, heat load calculations were performed for the building for summer and monsoon as presented in Tables 3 and 4 respectively.

**Table 3:** Heat load of the building considered for different cities during summer.

Name of the city	Room sensible heat (TR)	Room latent heat (TR)	Outdoor air heat (TR)	Total heat load (TR)	Grand total load (TR)
Ahmedabad	221.80	5.26	55.63	282.69	291.17
Bikaner	231.75	− 0.39	23.69	255.04	262.69
Chennai	205.79	11.88	88.88	306.54	315.74
Kolkata	200.16	15.19	107.77	323.12	332.81
Bangalore	187.61	4.85	19.99	212.45	218.82
Pune	204.49	13.12	96.74	314.34	323.77
New Delhi	221.36	3.06	39.05	263.47	271.37

**Table 4:** Heat load of the building considered for different cities during monsoon.

Name of the city	Room sensible heat (TR)	Room latent heat (TR)	Outdoor air heat (TR)	Total heat load (TR)	Grand total load (TR)
Ahmedabad	173.33	23.18	140.81	337.32	347.44
Bikaner	170.74	22.49	133.28	326.50	336.30
Chennai	178.53	22.63	141.72	342.87	353.16
Kolkata	179.82	24.70	158.12	362.64	373.52
Bangalore	149.10	17.11	73.21	239.43	246.61
Pune	152.85	17.39	78.81	249.06	256.53
New Delhi	172.47	23.32	140.99	336.78	346.88

In the process of conducting heat load calculations, the identical industrial shop floor as specified previously is assumed, maintaining its dimensions, occupancy and connected equipment load. Further, it is assumed that the indoor design specifications remain constant, while the outdoor design parameters are modified to evaluate the resultant heat load on the structure.

## Results and discussion

**Fig. 10:**
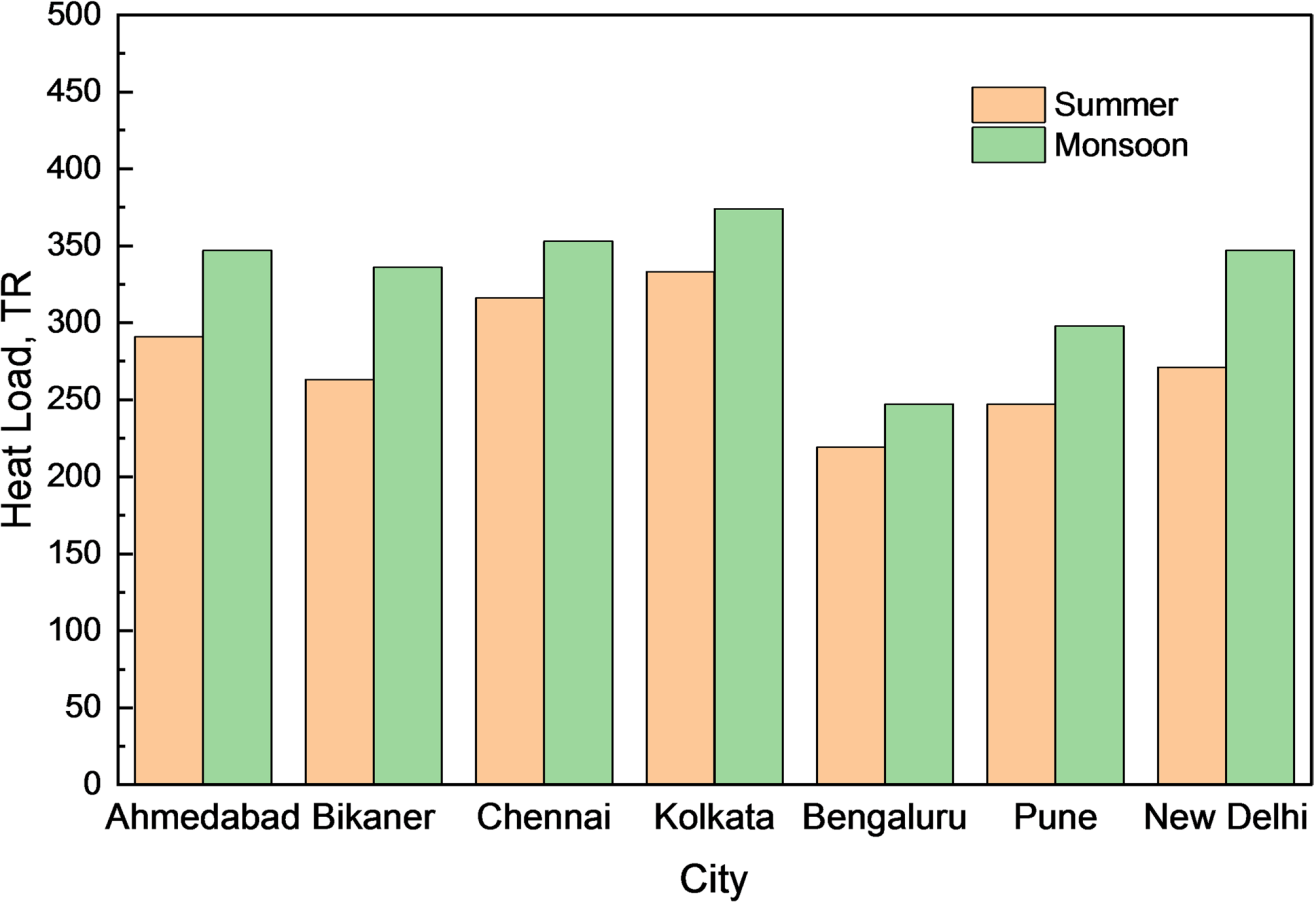
Comparison of heat load variations in various cities for summer and monsoon conditions.

The present study analyses cooling capacity requirements for three different cooling system configurations considering monsoon and summer heat load conditions at various Indian cities. The cooling system requirements were performed for an industrial building with specific heat load conditions being located in cities of India having varied climatic conditions. Tables 5 and 6 demonstrate the building heat loads in various cities based on the outdoor design ambient conditions for summer and monsoon climates, respectively. It is observed that during summer, the sensible heat load attains its maximum value than in monsoon. However, the total load values are higher across all cities during monsoon climatic conditions compared to summer as shown in Fig. 10. The reason for higher heat load during monsoon must be due to higher humidity in the air and latent heat than in summer.

**Table 5:** Actual outdoor conditions observed for the building in Chennai during monsoon.

Condition	Dry bulb temperature (°C)	Wet bulb temperature (°C)	Relative humidity (%)	Absolute Humidity (g/kg)	Enthalpy (kJ/kg)
Ambient	34.30	29.24	69	23.80	95.57
Required	26.00	19.70	55	11.88	56.30
Difference	8.30	9.54	14	12.08	39.27

**Table 6:** Performance of existing IEC system at Chennai during monsoon for actual building outdoor conditions.

Condition	Dry bulb temperature (°C)	Wet bulb temperature (°C)	Relative humidity (%)	Enthalpy (kJ/kg)
Ambient	34.30	29.24	69.00	95.57
IEC Outlet	31.01	28.50	82.90	91.87
Required	26.00	19.70	55.00	56.30
Load on chiller	5.01			35.57

### Cooling capacity requirements for IEC and modified IEC at Chennai

Initially, the cooling capacity requirements for IEC and Modified IEC configurations for the Chennai location were determined considering outdoor conditions during monsoon as shown in Table 5.

Heat load on the chiller for cooling and dehumidification of the air stream can be calculated by multiplying the mass flow rate of the air by the change in enthalpy.

2 $$Q = \dot{m} \times dh$$

where Q is the heat transfer rate of the air stream to be cooled, $\dot{m}$ is the mass flow rate of air and ‘dh’ represents the enthalpy difference. The smaller ‘dh’ represents a lower heat transfer rate thereby reducing the load on the chiller. The air stream first undergoes precooling through an existing indirect evaporative cooler before passing through the chiller coils to achieve the desired indoor conditions. For an airflow rate of 95,150 cfm (50.29 kg/s), the standalone chilled water system requires 589.65 TR, while using ambient air as working air lowers this to 534.09 TR and return air further reduces it to 503.76 TR, with the existing indirect evaporative cooling unit cutting chiller capacity to 55.56 TR and the modified system reducing it to 86.89 TR.

Based on the values in Table 5, results were generated for IEC and modified IEC cooling capacity requirements as depicted in Tables 6, 7 respectively for monsoon conditions. For the Indirect evaporative cooling system with ambient air as working air during monsoon, the wet bulb effectiveness of the system ranges from 40 to 80%. Further, the temperature reduction potential of the existing indirect evaporative cooler is 3.29 °C and reduces the load on the chiller to 5.01 TR for 35.57 kJ/kg of air stream as shown in Table 6. Similarly, in the modified indirect evaporative cooling system with return air as working air, the wet bulb effectiveness of the system was comparatively higher than the existing indirect evaporative cooling system. Due to this, the temperature reduction potential of the modified evaporative cooling system was better while the temperature reduction potential of the modified indirect evaporative cooler was calculated to be 5.01 °C. There is a reduction in the load on the chiller was observed for the modified indirect evaporative cooling system which is calculated to be 3.08 TR for 33.55 kJ/kg of air stream as shown in Table 7.

**Table 7:** Performance of modified IEC system at Chennai during monsoon for actual building outdoor conditions.

Condition	Dry bulb temperature (°C)	Wet bulb temperature (°C)	Relative humidity (%)	Enthalpy kJ/kg)
Ambient	34.30	29.24	69.00	95.57
IEC Outlet	29.08	28.07	92.70	89.85
Required	26.00	19.70	55.00	56.30
Load on chiller	3.08			33.55

**Fig. 11:**
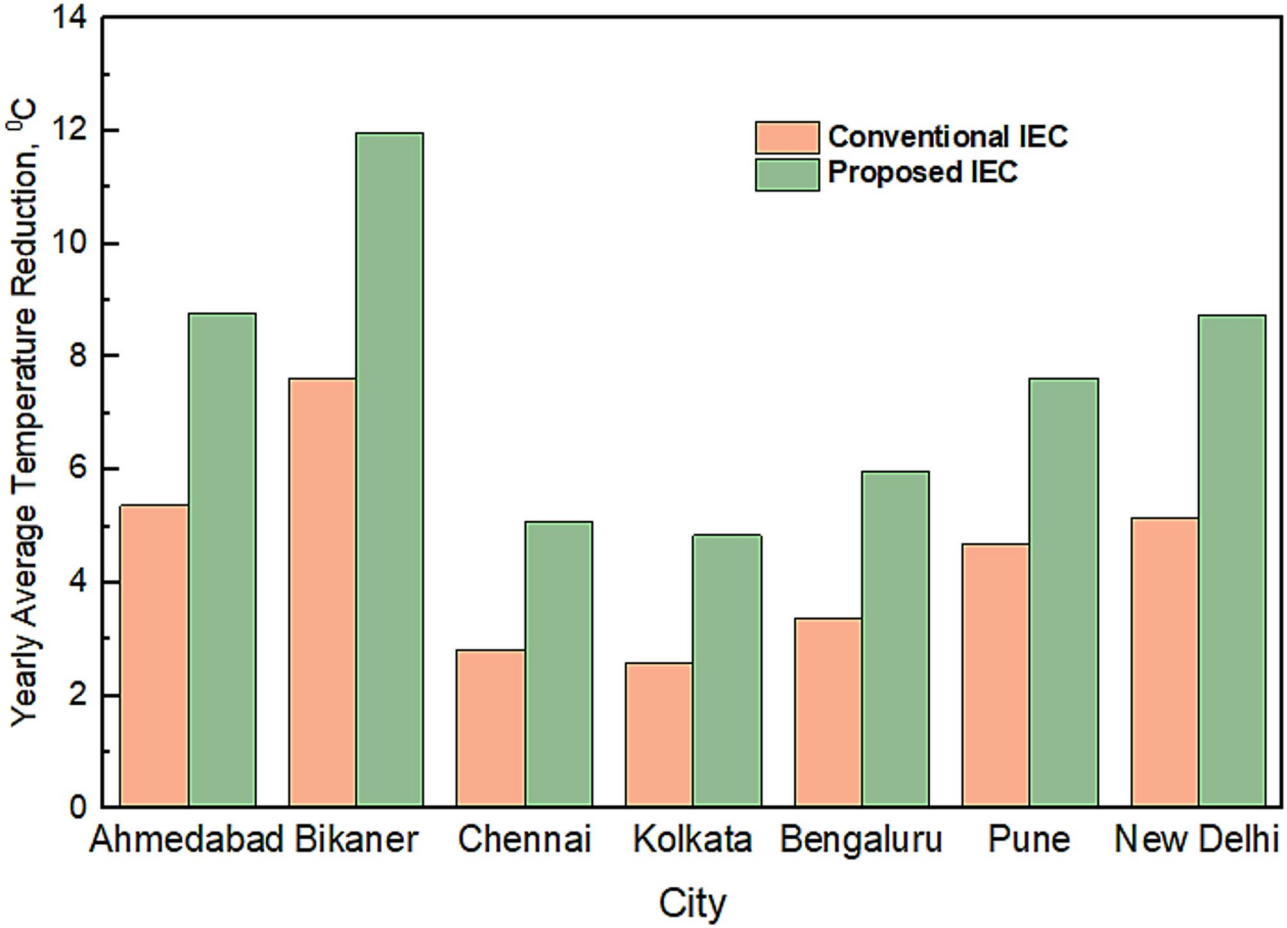
Comparison of Temperature reduction potential for conventional IEC and modified IEC at various cities.

In continuation to the analyses, the performance of IEC and modified IEC for other locations were calculated and compared as shown in Fig. 11. The modified IEC system performs significantly better than the existing IEC system in all climatic conditions. The average temperature reduction potential of both systems is maximum in hot and dry regions and minimum in warm and humid regions.

**Fig. 12:**
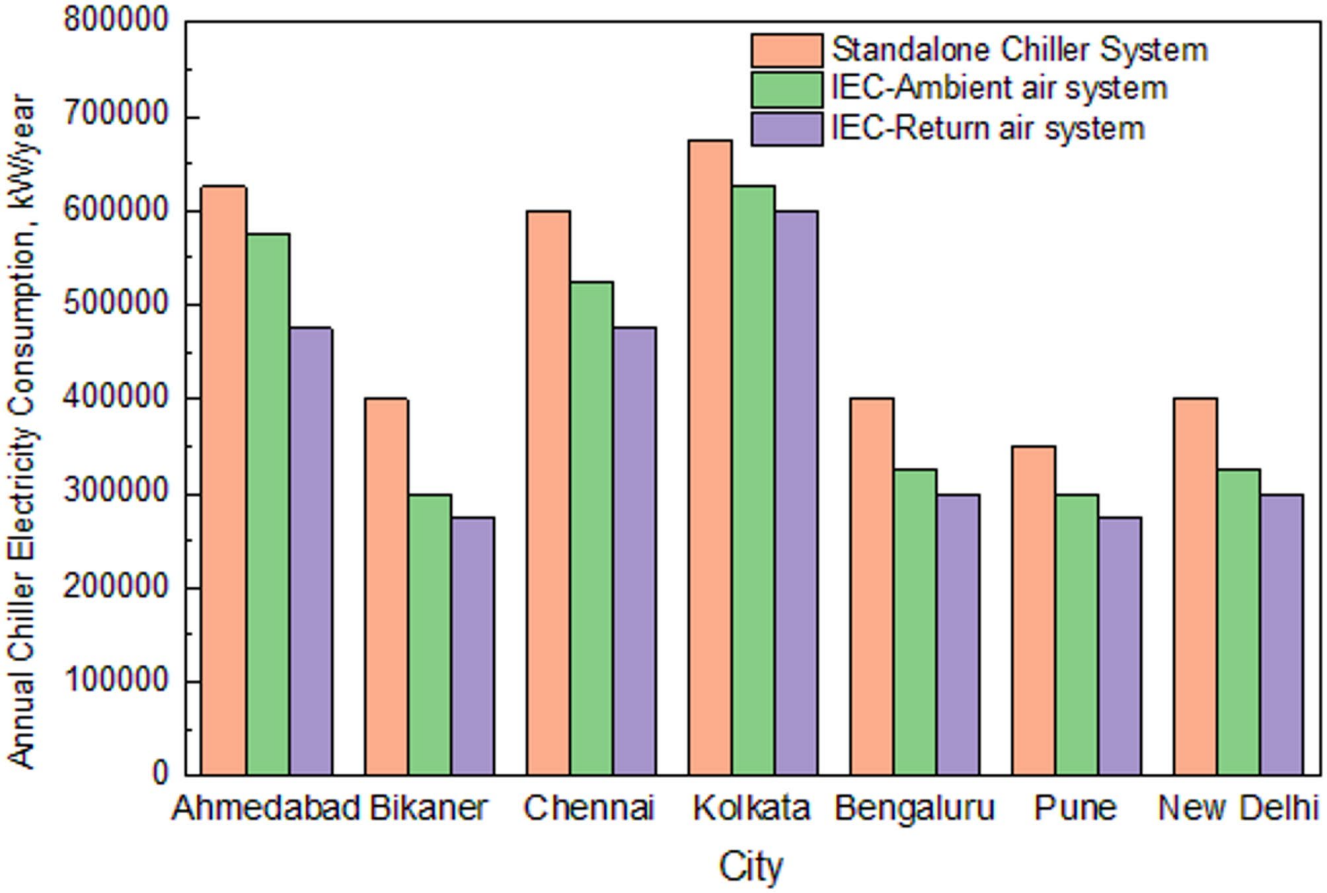
Comparison of Chiller electricity consumption at various cities for three cooling system configurations.

Finally, the cooling system electrical consumption at various cities for three configurations was calculated as presented in Fig. 12. The existing indirect evaporative cooling system performs better than the stand-alone chiller system and consumes 27.53% less electrical energy. The modified indirect evaporative cooling system consumes 34.97% less energy when compared to the stand-alone chiller system to handle the same heat load of the building.

## Conclusion

This study made a quantitative assessment of the performance of a hybrid multi-stage evaporative cooling-chiller system for large buildings in different Indian climates during summer and monsoon seasons. Three different system designs: stand-alone chiller, conventional IEC-chiller, and modified IEC-chiller with return air as secondary stream, were analyzed for comparison through psychrometric charts and building heat load calculations. The analysis clearly shows that the indirect evaporative pre-cooling effect results in a substantial reduction in chiller capacity in all considered climates. The conventional IEC-chiller design resulted in a capacity reduction of about 50–60 TR, while the modified IEC design resulted in a much higher capacity reduction of 80–140 TR, depending on the ambient conditions and the load characteristics. This directly affects the cooling capacity and capital costs of the HVAC system for buildings of similar size.

Results from energy performance analysis reveal that, for equal cooling demands, the traditional IEC-chiller system consumes 27.53% less electrical power than the stand-alone chiller system. Further enhancement is obtained using the modified IEC-return air system, which results in a 34.97% reduction in electricity consumption, thus validating the efficacy of return-air-assisted indirect evaporative cooling, especially in high-humidity monsoon climatic conditions, where the performance of conventional evaporative cooling is limited. The modified IEC system always provided greater cooling effectiveness and higher wet-bulb effectiveness in all climatic zones, with maximum benefits obtained in hot and dry climatic zones and sustained performance in humid monsoon climatic conditions. By transferring a substantial portion of the sensible cooling load from the chiller, the proposed system allows for improved part-load performance, reduced compressor operation, and improved system resilience during latent load-dominated periods.

In conclusion, the present investigation clearly establishes that return-air-assisted multi-stage evaporative cooling is a technically feasible and energy-efficient hybrid cooling technology for large commercial and industrial buildings in various Indian climatic conditions. The obtained results in terms of chiller capacity and electrical power consumption demonstrate its potential to reduce operational costs, peak electricity demand, and facilitate sustainable design of HVAC systems in regions experiencing rising cooling demands.

## Supplementary Information

Supplementary Information.

## Data Availability

The datasets analyzed during the current study are available from the corresponding author upon reasonable request.

## Abbreviations

CC: Cooling coil
COP: Coefficient of performance
DBT: Dry bulb temperature, °C
DEC: Direct evaporative cooling
DIEC: Direct–indirect evaporative cooling
h: Enthalpy, J/kg
HVAC: Heating, ventilation, and air conditioning
IEC: Indirect evaporative cooling
ISHRAE: Indian society of heating, refrigerating and air conditioning engineers
$\dot{m}$: Airflow rate, kg/s
Q: Heat transfer, W
RH: Relative humidity, %
T: Temperature
TR: Ton of refrigeration
w: Specific humidity, g/kg of dry air
WBD: Wet bulb depression, °C
WBT: Wet bulb temperature, °C
