# Supplementary material for: A comparative analysis of multi-stage evaporative cooling and conventional chillers for office-scale cooling loads
Source: Sci Rep. 2026 Apr 2;16:11244. doi: 10.1038/s41598-026-41650-9 (PMC13046834; doi:10.1038/s41598-026-41650-9)
Supplement: Supplementary file 1 — Supplementary Information. [file 41598_2026_41650_MOESM1_ESM.docx]

**Supplementary Material**

**Table A1. Heat Load Calculations for summer season at a location in Chennai**

| Location | | Season | Area | Height |
| --- | --- | --- | --- | --- |
| Chennai | | Summer | 45741 | 18 |
| Sensible Heat | DBT | RH | WBT | Gr/lb |
| Outside Condition | 101.12 | 36.00 | 77.90 | 107.10 |
| Inside Condition | 78.80 | 55.00 | 67.20 | 81.00 |
| Difference | 22.32 |  |  | 26.10 |
| ADP | 64.00 |  |  |  |
| Glass | Area (Sqft) | Sun Gain / Temp Diff (F) | U - Btu/Hr F Sqft | Btu/Hr |
| East Glass | 0.00 | 11.00 | 0.67 | 0.00 |
| West Glass | 0.00 | 164.00 | 0.67 | 0.00 |
| South Glass | 0.00 | 11.00 | 0.67 | 0.00 |
| North Glass | 0.00 | 11.00 | 0.67 | 0.00 |
| Skylight 10% of Roof | 4574.00 | 22.32 | 0.50 | 51045.84 |
| Walls | | | | |
| East Wall | 0.00 | 25.32 | 0.34 | 0.00 |
| West Wall | 5325.00 | 19.32 | 0.34 | 34978.86 |
| South Wall | 2840.00 | 23.32 | 0.34 | 22517.79 |
| North Wall | 2840.00 | 11.32 | 0.34 | 10930.59 |
| East Wall - GI Sheet with  Insulation | 0.00 | 25.32 | 0.20 | 0.00 |
| West Wall - GI Sheet with  Insulation | 3874.00 | 19.32 | 0.20 | 14969.14 |
| South Wall - GI Sheet with  Insulation | 2066.00 | 23.32 | 0.20 | 9635.82 |
| North Wall - GI Sheet with  Insulation | 2066.00 | 11.32 | 0.20 | 4677.42 |
| GI roof with Insulation | 45741.00 | 50.32 | 0.14 | 322236.20 |
| Transmission Gains | | | | |
| All Glass | 0.00 | 22.32 | 1.14 | 0.00 |
| Ceiling | 0.00 | 17.32 | 0.34 | 0.00 |
| Floor | 0.00 | 17.32 | 0.34 | 0.00 |
| Partition Wall | 0.00 | 17.32 | 0.34 | 0.00 |
| Internal Heat Gains | | | | |
| No of People | 150.00 |  | 255.00 | 38250.00 |
| Machinery Load | 380.00 | 0.35 | 3412.00 | 453796.00 |
| Light | 45.70 | 1.25 | 3412.00 | 195085.00 |
| Sub Total |  |  |  | 1158122.6 |
| Supply Fan & Duct Loss - 10% |  |  |  | 115812.27 |
| Room Sensible Heat |  |  |  | 1273934.9 |
| Air Changes Per Hour | 1.00 | 45741.00 | 18.00 | 13722.30 |
| CFM/Person | 5.00 | 150.00 |  | 750.00 |
| CFM/Sqft | 0.06 | 45741.00 |  | 2744.46 |
| Outdoor Air Heat CFM Bypass Factor 0.12 | | | | |
| Air Quantity | 15941 | 22.32 | 1.08 | 46113.37 |
| Effective Room Sensible Heat |  |  |  | 1320048 |

| Latent Heat |  |  |  | Btu/Hr |
| --- | --- | --- | --- | --- |
| No of People | 150 |  | 245 | 36750.00 |
| SA Fan & Duct Loss - 10% |  |  |  | 3675.00 |
| Room Latent Heat |  |  |  | 40425.00 |
| Outdoor Air Heat | | | | |
| 15941 | 26.1 | 0.12 | 0.68 | 33951.45 |
| Effective Room Latent Heat |  |  |  | 74376.45 |
| Outdoor Air Heat | | | | |
| 15941 | 22.32 | 0.88 | 1.08 | 338164.71 |
| 15941 | 26.10 | 0.88 | 0.68 | 248977.31 |
| Total Outdoor Air Heat |  |  |  | 587142.02 |
|  | | | | |
| Total Heat (Btu/Hr) |  |  |  | 1981566.77 |
| RA Duct/fan, CHW Pump Loss - 3% |  |  |  | 59447.00 |
|  | | | | |
| Grand Total (Btu/Hr) |  |  |  | 2041013.77 |
| Heat Load (TR) |  |  |  | 170.08 |
|  | | | | |
| Dehumidification Temp Rise (F) |  |  |  | 13.02 |
| Dehumidification Air Quantity  (CFM) |  |  |  | 93847.28 |
|  | | | | |
| CFM/TR |  |  |  | 551.77 |
| Sqft/TR |  |  |  | 268.93 |
| CFM/Sqft |  |  |  | 2.05 |
| Effective Sensible Heat Factor |  |  |  | 0.98 |

**Table A2. Heat Load Calculations for monsoon season at a location in Chennai**

| Location | | Season | Built Area | Height |
| --- | --- | --- | --- | --- |
| Chennai | | Summer | 45741 | 18 |
| Sensible Heat | DBT | RH | WBT | Gr/lb |
| Outside Condition | 89.78 | 82.90 | 83.12 | 161.70 |
| Inside Condition | 78.80 | 55.00 | 67.20 | 81.00 |
| Difference | 10.98 |  |  | 80.70 |
| ADP | 64.00 |  |  |  |
| Glass | Area Sqft | Sun Gain / Temp Diff (F) | U - Btu/Hr F Sqft | Btu/Hr |
| East Glass | 0.00 | 11.00 | 0.67 | 0.00 |
| West Glass | 0.00 | 164.00 | 0.67 | 0.00 |
| South Glass | 0.00 | 11.00 | 0.67 | 0.00 |
| North Glass | 0.00 | 11.00 | 0.67 | 0.00 |
| Skylight 10% of Roof | 4574.00 | 10.98 | 0.50 | 25111.26 |
| Walls | | | | |
| East Wall | 0.00 | 13.98 | 0.34 | 0.00 |
| West Wall | 5325.00 | 7.98 | 0.34 | 14447.79 |
| South Wall | 2840.00 | 11.98 | 0.34 | 11567.89 |
| North Wall | 2840.00 | -0.02 | 0.34 | -19.31 |
| East Wall - GI Sheet with  Insulation | 0.00 | 13.98 | 0.20 | 0.00 |
| West Wall - GI Sheet with  Insulation | 3874.00 | 7.98 | 0.20 | 6182.90 |
| South Wall - GI Sheet with  Insulation | 2066.00 | 11.98 | 0.20 | 4950.14 |
| North Wall - GI Sheet with  Insulation | 2066.00 | -0.02 | 0.20 | -8.26 |
| GI roof with Insulation | 45741.00 | 38.98 | 0.14 | 249617.79 |
| Transmission Gains | | | | |
| All Glass | 0.00 | 10.98 | 1.14 | 0.00 |
| Ceiling | 0.00 | 5.98 | 0.34 | 0.00 |
| Floor | 0.00 | 5.98 | 0.34 | 0.00 |
| Partition Wall | 0.00 | 5.98 | 0.34 | 0.00 |
| Internal Heat Gains | | | | |
| No of People | 150.00 |  | 255.00 | 38250.00 |
| Machinery Load | 380.00 | 0.35 | 3412.00 | 453796.00 |
| Light | 45.70 | 1.25 | 3412.00 | 195085.00 |
| Sub Total |  |  |  | 998981.19 |
| Supply Fan & Duct Loss - 10% |  |  |  | 99898.12 |
| Room Sensible Heat |  |  |  | 1098879 |
| Air Changes Per Hour | 1.00 | 45741.00 | 18.00 | 13722.30 |
| CFM/Person | 5.00 | 150.00 |  | 750.00 |
| CFM/Sqft | 0.06 | 45741.00 |  | 2744.46 |
| Outdoor Air Heat | CFM | Bypass Factor | 0.12 |  |
| Air Quantity | 15941 | 10.98 | 1.08 | 22684.80 |
| Effective Room Sensible Heat |  |  |  | 1121564 |

| Latent Heat |  |  |  | Btu/Hr |
| --- | --- | --- | --- | --- |
| No of People | 150 |  | 245 | 36750 |
| SA Fan & Duct Loss - 10% |  |  |  | 3675 |
| Room Latent Heat |  |  |  | 40425 |
| Outdoor Air Heat | | | | |
| 15941 | 80.7 | 0.12 | 0.68 | 104976.3246 |
| Effective Room Latent Heat |  |  |  | 145401.3246 |
| Outdoor Air Heat | | | | |
| 15941 | 10.98 | 0.88 | 1.08 | 166355.2218 |
| 15941 | 80.7 | 0.88 | 0.68 | 769826.3807 |
| Total Outdoor Air Heat |  |  |  | 936181.6025 |
|  | | | | |
| Total Heat (Btu/Hr) |  |  |  | 2203147.036 |
| RA Duct/fan, CHW Pump Loss -  3% |  |  |  | 66094.41108 |
|  | | | | |
| Grand Total (Btu/Hr) |  |  |  | 2269241.447 |
| Heat Load (TR) |  |  |  | 189.1034539 |
|  | | | | |
| Dehumidification Temp Rise (F) |  |  |  | 13.024 |
| Dehumidification Air Quantity  (CFM) |  |  |  | 79736.2781 |
|  | | | | |
| CFM/TR |  |  |  | 421.65 |
| Sqft/TR |  |  |  | 241.88 |
| CFM/Sqft |  |  |  | 1.74 |
| Effective Sensible Heat Factor |  |  |  | 0.90 |
